# Supplementary material for: Caterpillar Responses to Gustatory Stimuli in Potato Tuber Moths: Electrophysiological and Behavioral Insights
Source: Life (Basel). 2023 Nov 7;13(11):2174. doi: 10.3390/life13112174 (PMC10672149; doi:10.3390/life13112174)
Supplement: Supplementary file 1 [file life-13-02174-s001.zip › life-2676041-Supplementary.pdf]

Supplementary Materials for

Caterpillar Responses to Gustatory Stimuli in Potato Tuber Moths: Electrophysiological and Behavioral Insights

This document contains:

Figure S1. Gustatory SSR test of mouthparts and maxillary palp sensillum styloconicum of the 4th instar larvae *P. operculella*

Figure S2. Schematic diagram of feeding selection behavior of the 4th instar larvae *P. operculella*

Table S1. Lethal effects of the nicotine against the larvae of 4th instar larvae *P. operculella*

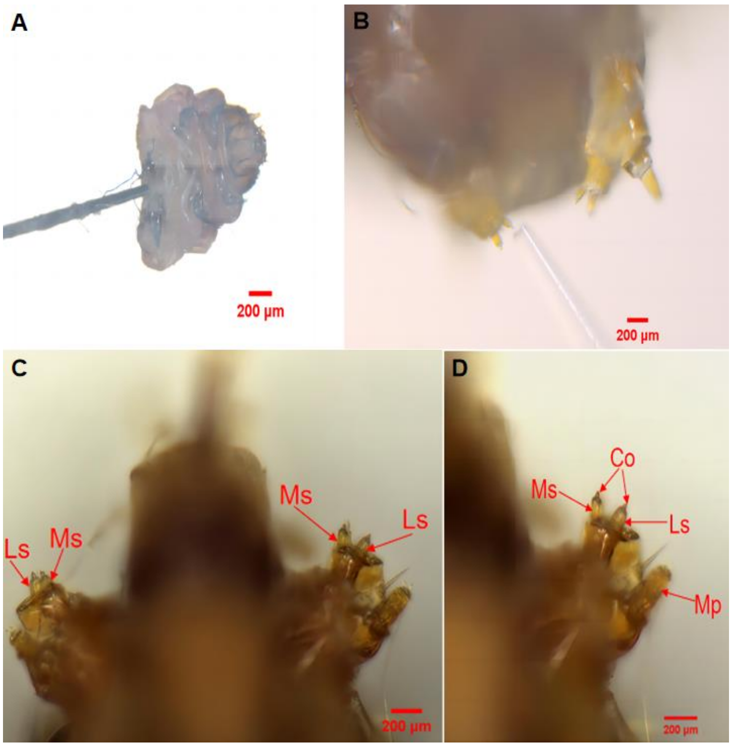

Figure S1. Gustatory SSR test of mouthparts and maxillary palp sensillum styloconicum of the 4th instar larvae *P. operculella*. A: Tungsten wire securing the 4th instar larva of the potato tuberworm. B: Glass capillary inserted into the larva's mouthparts at the styloconic sensillum end. Imaging was conducted using a stereomicroscope (DMC4500, Leica) and images were captured using LAS V4.9 software. C, D: Schematic representation of the styloconic sensilla on the labrum of the 4th instar larva of the potato tuberworm. Images of the larval mouthparts were obtained using a super depth-of-field 3D microscope (Smartzoom 5, Zeiss). MP: Maxillary palpus; Ls: Lateral sensillum styloconicum; Ms: Medial sensillum styloconicum; Co: Conic tip of the sensillum.

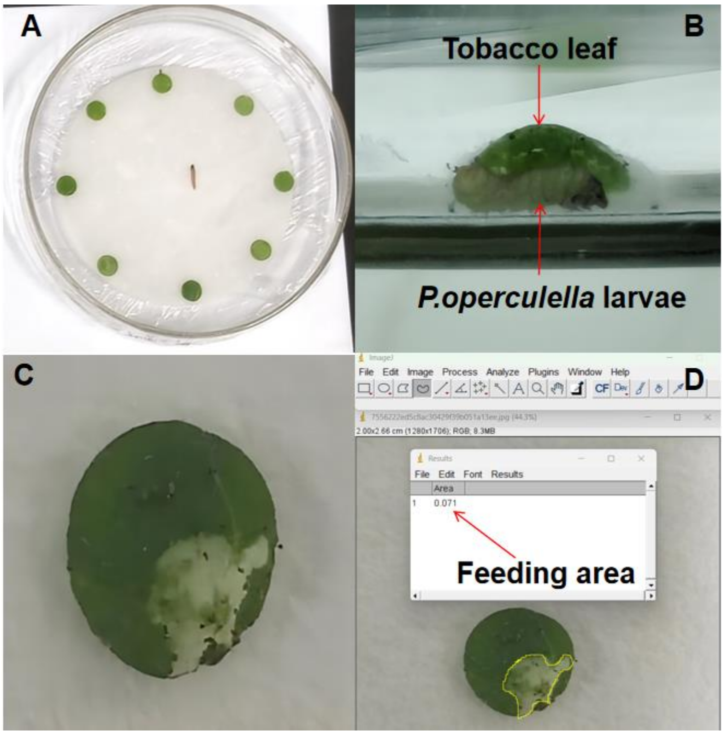

Figure S2. Schematic diagram of feeding selection behavior of the 4th instar larvae *P. operculella*. A: Dual-choice leaf disk assay. B: A larva was feeding on tobacco leaf disks by leaf diving. C: A tobacco leaf disk after feeding. D: Measuring the area of tobacco leaf disks taken using image J software.

Table S1. Lethal effects of the nicotine against the larvae of 4th instar larvae *P. operculella*.

Nicotine Concentration（mmoL/L）	Total Population（head）	Number of Dead（head）	Death Rate（%）
10	35	2	5.71%
30	35	5	14.29%
50	35	7	20.00%
